# Biomarkers for gastric cancer: Progression in early diagnosis and prognosis (Review)

**DOI:** 10.3892/ol.2015.2959

**Published:** 2015-02-12

**Authors:** ZILIANG JIN, WEIHUA JIANG, LIWEI WANG

**Affiliations:** Department of Oncology, Shanghai Key Laboratory of Pancreatic Diseases, Shanghai Jiaotong University Affiliated First People’s Hospital, Shanghai 200080, P.R. China

**Keywords:** gastric cancer, early diagnosis, prognosis, biological marker

## Abstract

Gastric cancer is one of leading causes of cancer-related mortality worldwide and is a notable disease due to its heterogeneity. Recently, numerous studies have investigated the molecular basis of gastric cancer, involving the alteration of pathogenesis, and invasion and metastasis. With the development of modern technologies, various novel biomarkers had been identified that appear to possess diagnostic and prognostic value; therefore, the present review describes our current knowledge of biomarkers for the early diagnosis and prognosis of gastric cancer. Classic biomarkers for gastric cancer diagnosis include carcinoembryonic antigen and cancer antigen 19-9, while microRNA and DNA hypomethylation are proposed as novel biomarkers. Excluding classical biomarkers, biomarkers for determining the progression and prognosis of gastric cancer focus on targeting microRNAs, epigenetic alterations and genetic polymorphisms.

## 1. Introduction

A total of 989,600 new cases of gastric cancer and 738,000 gastic cancer related mortlities are estimated to have occurred in 2008 worldwide, accounting for 8% of the total cases and 10% of total mortalies due to cancer worldwide (1). The incidence and mortality rates of gastric cancer have decreased overall in recent years, however, gastric cancer remains the leading cause of cancer-related mortality in developing countries (1). Despite progression in the diagnosis and treatment of advanced gastric cancer, the prognosis of gastric cancer patients remains poor, in part due to the low rate of diagnosis during its early stages. Numerous studies investigating the molecular mechanisms of gastric cancer invasion and metastasis have identified that survival is associated with the ability of the cancer to metastasize (2–4). Standard biomarkers used for gastric cancer diagnosis include carcinoembryonic antigen (CEA) and cancer antigen 19-9 (CA19-9) (5), however, recently microRNA and DNA hypomethylation have been proposed as novel biomarkers (6,7). TNM staging (tumor, lymph nodes and metastasis) was commonly used to assess patient prognosis in developed and developing countries. However, the approach was insufficient, because prognosis often varies between patients at the same tumor stage (8). The current review focuses on the currently available biomarkers for the early diagnosis and prognosis of gastric cancer.

## 2. Biomarkers for early diagnosis

### Classical biomarkers

#### CEA

CEA was initially identified in 1965 (9) and was first applied for the diagnosis of early gastric cancer in 1980 (10). CEA is currently regarded as the most valuable serum protein marker for identifying patients at risk of developing gastric cancer and for the diagnosis of early stage gastric cancer. However, serum CEA can be detected in patients with alternative types of carcinoma, thus, it exhibits low specificity and sensitivity.

#### CA 19-9

CA 19-9 has previously been a commonly used marker in gastrointestinal cancer; however, it is present in a number of types of cancer, in particular pancreatic and gastric cancer. The CA 19-9 test in combination with the CEA test is a useful adjunct for monitoring carcinoma of the stomach; however, the sensitivity of performing these assays concurrently is comparable to performing the CEA assay alone in gastric carcinoma (11).

### Novel biomarkers

#### microRNA (miR/miRNA)

In gastric cancer, 21 individual miRNAs and six miRNA clusters are consistently upregulated, whereas miR-29c, miR-30a-5p, miR-148a, miR-375 and miR-638 are typically downregulated (12–16). The biological functions of miRNAs in gastric cancer are involved in tumor formation and progression, affecting cell cycle progression, apoptosis, invasion and metastasis (12,13,17–23). However, the application of these miRNAs as diagnostic or prognostic biomarkers poses a challenge.

Numerous studies have demonstrated that miRNAs are stable and detectable in human plasma, thus, indicating their potential as biomarkers for the early diagnosis of gastric cancer. Cai *et al* (24) measured the plasma expression levels of 15 selected miRNAs, including miR-106b, miR-20a and miR-221, and identified a statistically significant elevation in expression levels in gastric cancer patients. Furthermore, Zhang *et al* (25) identified that the expression levels of miR-421 in gastric cancer were significantly different compared with in benign gastric diseases, markedly improving upon the detection of early gastric cancer by using serum CEA alone. Yu *et al* (26) additionally determined miR-129 to be a potential biomarker for the screening of gastric cancer.

#### DNA hypomethylation

The epigenetic phenomenon of cancer, particularly gastric cancer, is mainly dependent on alterations in DNA methylation pattern. Global DNA hypomethylation is an early molecular event in histone protein-associated gastric carcinogenesis (27). Oishi *et al* (28) confirmed that the silencing of Sox17 frequently occurs in early gastric cancer, therefore, hypermethylation of the Sox17 gene may be applied as a useful molecular diagnostic marker in early gastric cancer (Table I).

## 3. Prognostic biomarkers

### Classical prognostic factors

#### Microsatellite instability (MSI)

MSI, resulting from errors in DNA replication, is characteristic of hereditary types of gastric cancer (29). MSI can be divided into high-level, low-level or microsatellite-stable, according to the mutation frequency. A high frequency of MSI is the result of epigenetic inactivation of the mismatch repair gene human mutL homolog 1, whereas mutations in the transforming growth factor-β (TGF-β) receptor II (RII), insulin-like growth factor-IIR and B-cell lymphoma-2 (Bcl-2)-associated X protein genes in sporadic gastric cancer are associated with a decreased propensity for invasion and nodal metastasis (30–33). Numerous studies examining the association between MSI and the prognosis of gastric cancer have been performed, the majority of which have demonstrated that MSI is associated with less aggressive behavior and more favorable survival; however, additional studies have indicated no prognostic value for MSI (34–36).

#### Growth factor signaling pathways and cytokines

##### i) Growth factors

Gastric cancer cells express a variety of growth factors and their corresponding receptors, including epidermal growth factor/receptor (EGF/R) (37), TGF-β/R and vascular endothelial cell growth factor/receptor (VEGF/R), fibroblast growth factor/receptor (bFGF/R), and platelet-derived endothelial cell growth factor/receptor (PD-ECGF/R), which are involved in tumor cell growth, angiogenesis, invasion and proliferation, respectively (37,38).

EGFR and its homolog c-erbB-2 (HER2) are membrane receptors that are overexpressed in a variety of types of solid human cancer and are associated with a poor prognosis. García *et al* (39) identified a wide range of membranous EGFR and cytosolic HER2 expression levels in gastric cancer, which appears to be associated with the biological heterogeneity of these tumors. In addition, high tumor EGFR and HER2 expression levels were associated with an unfavorable outcome in patients with resectable gastric cancer.

However, numerous studies have indicated that HER2 is not a prognostic factor in gastric cancer. For example, Terashima *et al* (40) identified that overall and relapse-free survival rates were significantly lower in EGFR-positive patients compared with EGFR-negative patients, but similar in HER2-positive and -negative patients. Furthermore, Zhou *et al* (41) determined that the overexpression/amplification of HER-2/neu was not an independent predictor of survival in curatively resected gastric cancer patients.

Additional growth factors/receptors were determined to be associated with the prognosis of gastric cancer. For example, Kim *et al* (42) evaluated the expression of various growth factors/receptors in gastric adenocarcinoma, including EGFR, VEGF, VEGF-D, VEGFR-2, VEGFR-3, TGF-α, TGF-β1 and TGF-β RII, using a log rank test to identify that VEGF-D and VEGFR-2 expression was associated with patient survival, and Cox regression analysis to determine that advanced stage gastric cancer and positive expression of VEGF-D were poor prognostic factors. Thus, in gastric adenocarcinoma, VEGF-D expression levels may be of prognostic value, whereas the expression levels of the EGFR and TGF family may have only a minor effect. Furthermore, high expression of the FGFR4 protein may be associated with a poor prognosis in advanced gastric cancer patients via the acceleration of disease progression (43).

In gastric cancer, the mechanism of liver metastasis may be associated with the high frequency of c-Met overexpression in the carcinoma cells; therefore, the analysis of c-Met expression levels may be a useful indicator of liver metastasis in gastric cancer patients (44). Graziano *et al* (45) identified that ~10% of Caucasian gastric cancer patients harbored a MET gene copy number of five or above, and that this was significantly associated with an unfavorable prognosis. This data is of particular relevance to the current clinical development of anti-MET therapeutic compounds. Additionally, upregulation of the PIM-1 oncogene may be a prognostic tumor marker for gastric cancer, as PIM-1 overexpression in gastric glands has been shown to correlate with the formation of lymph node metastases and survival (46).

##### ii) Cytokines

Gastric cancer tumors produce various types of cytokine, including interleukin (IL)1, IL6, IL10, IL11, tumor necrosis factor (TNF), C-X-C motif chemokine (CXC)12, chemokine (C-C motif) ligand 1 and CXC receptor 2 (47–53). No mutations have been detected in key positions of glycoprotein 130 (gp130) in human gastric adenocarcinoma samples; however, gp130 activation may occur due to increased expression levels of the IL-6 and IL-11 cytokines, which have potential as valuable biomarkers (54). IL-6 induces AGS gastric cancer cell invasion via activation of the cellular-Rous sarcoma/ras homolog family member A (RhoA)/Rho-associated, coiled-coil-containing protein kinase signaling pathway, and RhoA expression may be a potential prognostic factor in gastric adenocarcinoma patients (55). Additionally, IL-12-positive cell density may be a significant independent prognostic factor in the analysis of advanced gastric cancer surgical specimens (50); and chemokine (C-C motif) receptor 4 and its ligands appear to be associated with increased tumor recurrence and impaired overall survival in gastric cancer patients (56).

#### Cell cycle factors and apoptosis

##### i) Cell cycle regulators

Abnormalities in cell-cycle regulators are associated with various aspects of gastric cancer, including cancer cell proliferation. Furthermore, specific cell cycle factors are associated with the prognosis of gastric cancer (57), for example, cyclins condition the progression of the cell cycle by activating appropriate serine-threonine kinases. Therefore, changes in cyclin expression levels lead to pathologies of cell division, including neoplastic proliferation. The activity of cyclins D1 and E when complexed with appropriate cyclin-dependent kinases may be inhibited by protein p21 (WAF1/CIP1), which functions as an inhibitor of the cell growth cycle (58). Of the two types of cyclin evaluated, only cyclin E is considered to be a significant regulatory factor and useful prognostic parameter in gastric cancer; furthermore, it has been demonstrated that reduced p27 expression is a negative prognostic factor for patients with cyclin E-positive tumors (59–61). Similarly, alterations in the p53 gene are associated with less favorable prognoses in advanced gastric cancer; this may be by the potential provision of vertical growth into the gastric wall. Multivariate analysis demonstrated that the overexpression of p53 was an independent prognostic factor for advanced gastric cancer patients (62), however, the expression of p53 alone exhibited no prognostic value for early gastric cancer patients (63). Additionally, the poor prognosis for patients with low retinoblastoma protein (pRb) expression levels and decreased pRb expression in lymph node metastases indicate that Rb and its associated genes may affect cancer progression (64).

##### ii) Apoptosis-associated factors

Apoptosis is an established type of programmed cell death. Abnormalities in apoptosis promote gastric carcinogenesis (65) and numerous apoptosis-associated factors have been determined to be prognostic indicators of gastric cancer (66–73). For example, the Bcl-2 proto-oncogene is important in determining the susceptibility of tumor cells to apoptosis; with Bcl-2 expression and a high apoptotic fraction determined to be important prognostic factors of survival in advanced gastric cancer patients (67,68). Fas (apoptosis-1/cluster of differentiation 95), a member of the TNF/nerve growth factor receptor superfamily, mediates apoptosis as a response to agonistic antibodies or Fas ligand (FasL) binding. Recently, it was reported that tumor cells are able to express FasL, inducing apoptosis in tumor-infiltrating lymphocytes and thus allowing them to escape host immune surveillance. In gastric carcinoma, tumor progression via the lymphatics is often observed, and lymph node metastasis is a critical factor affecting the recurrence and prognosis of cancer; previous studies have demonstrated that upregulation of FasL may correlate with this progression of gastric carcinoma (69,70). In addition, survivin is a recently characterized gene and a member of the inhibition of apoptosis family that inhibits apoptosis via pathways that do not involve the Bcl-2 family (74–76). Specific studies have indicated that survivin is present in the majority of gastric cancer cells, however, the nuclear localization of survivin appears to be physiologically important in hindering tumor progression (77), therefore, survivin may be an important predictive and prognostic parameter of poor outcome in gastric carcinoma (78).

### Novel prognostic factors

#### miRNA

miRNAs may be used as prognostic factors, as they are expressed in stable and robust levels in tissues and the circulation (74–76). In particular, miRNAs have been associated with survival times and disease stage in gastric cancer patients, as well as with tumor recurrence and metastasis to the lymph nodes.

Ueda *et al* (15) identified that low let-7 g and miR-433 expression levels, and high miR-214 expression levels were associated with unfavorable overall survival outcomes, independent of clinical covariates, including invasion depth, lymph-node, tumor stage and metastasis. An additional study confirmed that high miR-20a, miR-25, miR-93, miR-103, miR-106a, miR-106b, miR-130, miR-155, miR-221 and miR-222 expression levels in advanced gastric cancer tissues appeared to be risk factors highly associated with the penetration of the tumor through the serosa, distant metastasis, lymph node metastasis and poor long-term survival in those patients undergoing radical resection procedures and adjuvant systemic chemotherapy (79).

Circulating miRNA, which is stable and easy to obtain, was previously considered to be a promising biomarker for predicting survival in gastric cancer. For example, Komatsu *et al* (74) identified that a high miR-21 concentration in plasma was an independent prognostic factor in gastric cancer, and another study detected increased miR-200c levels in the blood of gastric cancer patients, indicating that miR-200c may be also a potential predictor of gastric cancer progression and survival (80). However, these studies of miRNAs as prognostic factors involved small sample sets; thus, validation in larger, independent cohorts is required prior to the application of miRNA assays in a clinical setting.

#### Epigenetic alterations

Genetic and epigenetic mechanisms are involved in molecular alterations and pathway dysregulation. In contrast to genetic alterations, epigenetic changes, such as DNA methylation and histone modifications, affect the phenotypic outcomes of a genome without changing the underlying DNA sequences (81).

The hypermethylation of CpG islands is associated with the silencing of tumor suppressor genes and is involved in tumorigenesis. The hypermethylation of various genes, such as cadherin 1 (CDH1) (82), coiled-coil domain containing protein 67 (83), methylated in tumors 31 (84), p16 (85), runt-related transcription factor 3 (85), E-cadherin (86), hMHL1 (87) and wingless-type MMTV integration site family, member 5A (88), has been identified to be associated with the prognosis of gastric cancer. However, not all methylation acts as a prognostic marker. Raf kinase inhibitory protein (RKIP) has been identified to be a member of a novel molecular class that has been indicated to be involved in cancer progression and the suppression of metastatic tumor spread; therefore, hypermethylation and loss of RKIP expression may be used as a marker to predict the clinical outcome of advanced gastric cancer (89). Similarly, methylated CDH1 predicts a poor prognosis in gastric cancer patients (82); however, S100A6 is important in the progression and prognosis of gastric cancer, and is upregulated by epigenetic regulation (90). Methylation of Bcl-2/adenovirus E1B 19-kDa-interacting protein 3 and death-associated protein kinase can predict a reduced response to chemotherapy and a poor prognosis in gastric cancer (91). Furthermore, paired box 5 (PAX5) is a novel functional tumor suppressor in gastric carcinogenesis, and detection of methylated PAX5 can be utilized as an independent prognostic factor in gastric cancer (92).

#### Genetic polymorphisms

Genetic polymorphisms appear to be an important cause of gastric carcinogenesis, with genetic susceptibility associated with gastric cancer risk. Various studies have demonstrated that the presence of numerous single nucleotide polymorphisms (SNPs) is associated with increased gastric cancer susceptibility; for example, excision repair cross-complementation group 1, CDH1, IL-10, IL-1, IL-6, VEGF and FAS gene SNPs have been found to be associated with the risk of gastric cancer (Table II) (93–101).

Additionally, specific SNPs appear to predict the outcome of chemotherapy or adjuvant chemotherapy treatment strategies. For example, polymorphisms of rs1801159 in dihydropyrimidine dehydrogenase (DPD), a key enzyme involved in the catabolism of 5-fluorouracil (5-FU), may be utilized as valuable FU-based chemotherapy response predictors for patients with gastric cancer in the Chinese population (102). Furthermore, the G/G genotype of the VEGF-634 G/C polymorphism is associated with higher serum expression levels of VEGF and a poor clinical outcome in patients with advanced gastric cancer who are treated with oxaliplatin, 5-FU and leucovorin (103). In addition, polymorphisms of glutathione *S*-transferase P1, xeroderma pigmentosum group D and X-ray repair cross complementing group 1 have been shown to predict the clinical outcome of gastric cancer patients to oxaliplatin/5-FU-based chemotherapy (104,105). The tumor protein p53 (TP53) codon 72 SNP was determined to be predictive of the response to chemotherapy, and correlated with the time to progression in advanced gastric cancer patients treated with paclitaxel and cisplatin chemotherapy (106).

Furthermore, various SNPs were associated with the prognosis of gastric cancer. Shirai *et al* (107) determined that the p53 SNP Arg72Pro was associated with a poor prognosis in gastric cancer (107), while Kim *et al* (106) identified that the TP53 codon 72 SNP was predictive of the chemotherapy response and that it was associated with the time to progression in advanced gastric cancer patients treated with paclitaxel and cisplatin chemotherapy. A different study determined that the TP53 codon 72 polymorphism was associated with gastric cancer patient survival in those treated with 5-FU-based post-operative chemotherapy (108); thus, the TP53 codon 72 polymorphism may be a potential prognostic factor in gastric cancer.

Numerous studies have been conducted to investigate the effect of cytokines and other pro-inflammatory mediator gene polymorphisms on the prognosis of gastric cancer, however, controversy remains. For example, García-González *et al* (109) determined that pro- and anti-inflammatory cytokine gene polymorphisms, such as IL1B, TNFA, LTA, IL6, IL12p40, IL4, IL1RN, IL10 and TGFB1, may not be relevant in determining the prognosis of gastric adenocarcinoma patients. Similarly, Liu *et al* (110) identified that IL-10 gene promoter polymorphisms may not be associated with the prognosis of advanced gastric cancer. By contrast, Tahara *et al* (111) proposed that the IL-1β-31CC and IL-1β-511TT genotypes, and the TNF-α-857T carrier may exhibit a protective effect against gastric cancer progression.

## 4. Conclusion

Gastric cancer is a noteworthy disease due to its heterogeneous properties. Although the molecular basis of gastric cancer has been thoroughly investigated, resulting in significant progression within the field, ubiquitous biomarkers are rare. Therefore, the identification of novel and specific markers to diagnose and predict gastric cancer survival is important.

With the development of modern technologies, such as genome and exome sequencing, and miRNA microarrays, various novel biomarkers with diagnostic and prognostic value have been identified. Although complicated, clinical trials for the screening of novel biomarkers prior to clinical application have been necessary, and thus far have resulted in promising outcomes.

## Figures and Tables

**Table I:** Major molecular markers associated with the early diagnosis of gastric cancer.

Molecule	Alteration	References
Classical biomarker		
CEA	Increase	10
CA19-9	Increase	11
miRNA		
miR-106b, miR-20a, miR-221, miR-421, miR-129	Upregulation	12–16,24–26
DNA hypomethylation		
Sox 17	Downregulation	28

CEA, carcinoembryonic antigen; CA19-9, cancer antigen19-9; miR/miRNA, microRNA.

**Table II:** Major molecular markers associated with the prognosis of gastric cancer.

Marker	Alteration	References
MSI	High level	30–36
Growth factors		
EGFR, HER-2, VEGF, TGF, c-MET	Overexpression	39–44
Cytokines		
IL-6, IL-11	Upregulation	54,55
Cell cycle regulators		
Cyclin E	Overexpression	59–61
Apoptosis-associated factors		
Bcl-2, Fas, survivin	Overexpression	67–70
miRNAs		
Let-7g, miR-433	Downregulation	15
miR-214, miR-21	Upregulation	15,77
Epigenetic alterations		
Runx3, E-cadherin, WNT5A	Hypermethylation	83–85
Genetic polymorphisms		
p53, IL-1, IL-10	SNP	104–106

MSI, microsatellite instability; EGFR, epidermal growth factor receptor; HER-2, human epidermal growth factor receptor-2; VEGF, vascular endothelial growth factor; TGF, transforming growth factor β; IL, interleukin; Bcl-2, B-cell lymphoma-2; miR/miRNA, microRNA; Runx3, runt-related transcription factor 3; WNT5A, wingless-type MMTV integration site family, member 5A; SNP, single-nucleotide polymorphism.
